# An automated controlled-release device for intravaginal hormone delivery

**DOI:** 10.3168/jdsc.2020-18816

**Published:** 2020-09-02

**Authors:** M. Masello, Y. Ren, D. Erickson, J.O. Giordano

**Affiliations:** 1 Department of Animal Science, Cornell University, Ithaca, NY 14853; 2 Sibley School of Mechanical and Aerospace Engineering, Cornell University, Ithaca, NY 14853

## Abstract

Our objective was to develop and validate an electronically controlled hormone-delivery device for reproductive control of cattle. After development and in vitro testing of a prototype device for intravaginal (IVG) hormone release, we aimed to demonstrate the feasibility of inducing luteal regression by automated treatment with PGF_2α_. The IVG device comprises an outer 3D-printed plastic housing, fluid reservoirs connected to delivery pumps and tubing, a programmable circuit board, and a retention mechanism. For in vitro testing, 4 pumps were programmed to release different target volumes (0.1, 0.2, 0.5, 1.0, and 2.0 mL) in 4 replicates (n = 80). A Bland-Altman plot was constructed to assess the magnitude of disagreement between expected and delivered volumes. Observations fell within acceptable limits of agreement (1.96 standard deviations) >95% of the time, indicating overall good agreement (mean difference = −0.005 mL). To assess in vivo performance of the IVG device, lactating Holstein cows with at least 1 corpus luteum ≥15 mm in diameter were randomly allocated to 1 of 3 treatments: (1) IM-PGF (n = 6): two 25-mg intramuscular doses of PGF_2α_ 24 h apart; (2) DEV-PGF (n = 6): four 25-mg doses of PGF_2α_ released automatically by the IVG device at 10- or 12-h intervals; and (3) DEV-CTL (n = 4): insertion of an empty IVG device (placebo control). Blood samples were collected at 0, 12, 24, 36, 48, and 72 h after treatment. Data were analyzed by ANOVA with repeated measures. All devices (10/10) remained in situ until removed at 48 h. Progesterone (P4) concentrations from 0 to 72 h were affected by treatment, time, and their interaction. Concentrations of P4 did not differ at time 0 but differed from 24 to 72 h: cows in IM-PGF and DEV-PGF had lesser P4 than cows in DEV-CTL. Conversely, P4 did not differ for IM-PGF and DEV-PGF during the experiment. We conclude that the current IVG hormone-releasing device prototype can be programmed to automatically release PGF_2α_ for successful induction of luteal regression in lactating dairy cows.

## Highlights

• We developed and validated a fully automated intravaginal hormone-delivery device.
• Our prototype intravaginal hormone-delivery device successfully induced luteal regression after automated release of PGF2?.
• Our automated hormone-delivery device may be an alternative tool to current methods for hormone administration.

## Graphical Abstract

**Figure:**
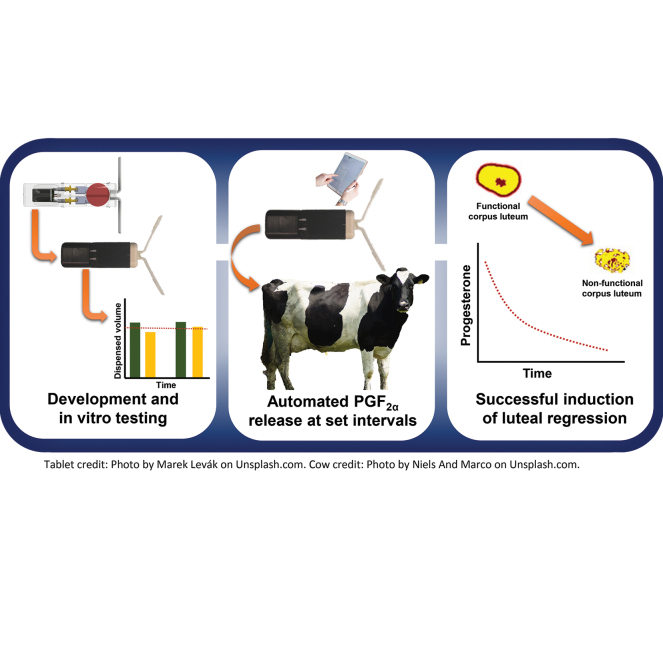
**Summary:** We developed and validated an electronically controlled hormone-delivery device for reproductive control of cattle and demonstrated the feasibility of inducing luteal regression by automated treatment with prostaglandin F2α. On-farm use of this automated delivery system might simplify herd management, reduce animal disruption, and enable improved ovulation synchronization protocols.

Timely and successful insemination of cows after they become eligible for pregnancy or a failed insemination is imperative to achieve optimal herd reproductive performance (Lamb et al., 2010; Wiltbank and Pursley, 2014). Therefore, a method used by many commercial farms to submit cows for insemination consists of synchronizing ovulation followed by timed AI (**TAI**; Pursley et al., 1995, 1997). Benefits of TAI include insemination at a predetermined time regardless of expression and detection of estrus and the possibility of achieving similar or greater fertility than that of AI at detected estrus (Pursley et al., 1997; Santos et al., 2017). A major caveat of implementing TAI programs is the need to administer multiple hormonal treatments as intramuscular (**IM**) or subcutaneous injections. This problem is exacerbated as novel and more complex protocols are developed to maximize fertility: in larger herds, more cows need to be synchronized at the same time and farms may lack critical resources to facilitate protocol implementation (e.g., dairy herd management software, proper facilities). Implementation of TAI protocols also requires significant human intervention and cow manipulation, which not only represents a cost burden for farms, but may also affect cow natural behaviors and time budgets (Bolinger et al., 1997).

Thus, a potential strategy to reduce the burden of implementing synchronization of ovulation protocols is to develop an all-encompassing delivery system for releasing all hormones of interest in the sequence, pattern, and dose required to synchronize ovulation. A requirement for successful synchronization of ovulation with an automated device is releasing hormones of interest at predefined time intervals at a rate and amount that elicit the desired physiological response. This is critical for reproductive hormones such as PGF_2α_ and GnRH, which exert their biological effects (i.e., LH surge for GnRH and luteal regression for PGF_2α_) by reaching target tissues in the form of sudden short-lived surges or pulses (i.e., minutes or a few hours) rather than in a sustained manner with elevated levels for prolonged periods (i.e., many hours or days). Another important consideration for the development of automated hormone-delivery devices is placement within or on the cow. Among all locations available for device placement and with potential to allow device functionality, the vagina offers unique benefits. Specifically, ease of insertion and removal, protection from damage from facilities and other animals, constant temperature, suitability for extended retention, and efficacy of reproductive hormones after intravaginal (**IVG**) delivery (Wijma et al., 2016, 2017; Masello et al., 2020). Although previous efforts were made to develop some electronically controlled IVG hormone-delivery devices (Cross et al., 2004; Künnemeyer et al., 2004), there is limited information about their performance and suitability for synchronization ovulation in cattle.

Therefore, our overall objective was to develop a fully automated hormone-delivery device for reproductive control of cattle and conduct proof-of-concept in vitro and in vivo validation studies. For the in vitro validation, our hypothesis was that assembled automated intravaginal hormone-delivery devices would be able to accurately release the amount programmed at 12-h intervals with <10% error. Thus, our objective was to demonstrate the precision of the device when programmed to release a fixed amount of fluid at specific time points. For the in vivo validation, we aimed to demonstrate complete luteal regression by automated delivery of PGF_2α_ because we have recently demonstrated similar luteal regression risk after IVG or IM administration of PGF_2α_ (Masello et al., 2020). We hypothesized that automated delivery of PGF_2α_ by an electronically controlled IVG device would induce luteal regression and that the changes in circulating P4 would be similar to those observed after IM injection of PGF_2α_.

The IVG device (Figure 1) comprises an outer 3D-printed plastic housing (12 × 4.0 × 3.0 cm), 2 silicone hormone reservoirs (∼5 mL) connected to delivery pumps (n = 2; Takasgo Fluidic Systems, Westborough, MA), a printed circuit board (**PCB**) powered by a rechargeable battery, and a retention mechanism. The circuit board is programmed in C language to deliver target doses at a scheduled time. Two general-purpose input/output (GPIO) pins are routed and programmed to control the on/off switch of the n-channel MOSFETs (metal–oxide–semiconductor field-effect transistor; Diodes Inc., Plano, TX), which controls the power supply for each peristaltic pump. In the “on” setting, liquid solution is pumped out of the hormone reservoirs through tubing that opens up to the exterior at the middle section of the device. Plastic elbows attached to each of the 2 orifices ensure proper liquid flow to the exterior of the device. Conformal urethane coating (M. G. Chemicals, Surrey, BC, Canada) of the PCB was performed to prevent moisture from reaching the electrical components. To ensure ease of insertion and minimize irritation of the vaginal mucosa, the device was coated with skin-safe silicone rubber (Dragon Skin FX-pro, Smooth-on Inc., Macungie, PA).

**Figure 1:**
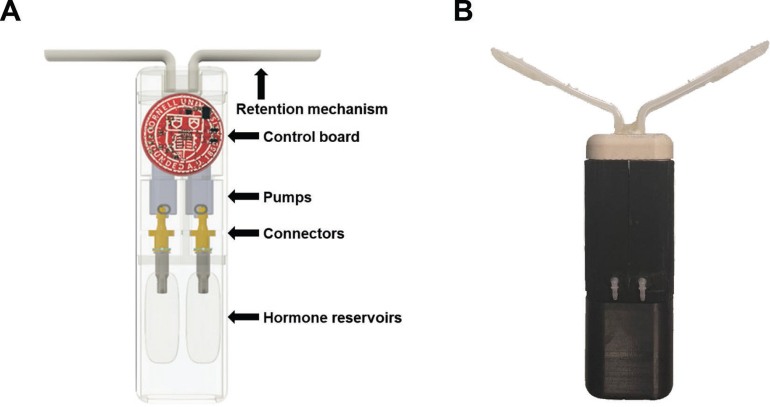
(A) Intravaginal device overview and internal structures; (B) sample device used in the current experiment.

Once the final prototype was assembled, the delivery rate for each pump was determined and a simulation of a 2-d in vivo test was conducted. As the pumps do not have the exact same internal release rate due to minor differences in assembly, we estimated the dispensing rate of each pump. Hormone reservoirs were loaded with 5 mL of distilled water and allowed to release at their own internal rate for 300 s over a precision scale (Radwag USA LLC, Miami, FL) at room temperature. The cumulative amount released per second over the 300-s period was measured and plotted (Figure 2A). Once the average release rate of each pump was defined, we calculated the time interval required to release a total of 0.1, 0.2, 0.5, 1.0, and 2.0 mL of water for each pump (n = 4) and programmed them to perform accordingly in 4 replicates (n = 80 doses); results are shown in Figure 2B. The volume delivered was calculated from the weight difference before and after release. To assess the magnitude of disagreement between the expected and delivered amounts and facilitate the detection of trends, a Bland-Altman plot (MedCalc, MedCalc Sofware bvba, Ostend, Belgium) was used to determine differences between target and delivered dose (y-axis) against target dose (x-axis). For doses in the 0.1- to 1.0-mL range, all observations fell within the limits of agreement, whereas one observation from pump 1 (difference = −0.06 mL, equivalent to 3% of target dose) and one observation from pump 2 (difference = 0.03 mL, equivalent to 1.5% of target dose) fell outside the limits of agreement for the 2.0-mL dose (Figure 2B). The overall difference between target and actual dose averaged −0.005 mL, which is less than a drop of water (0.05 mL), and indicated overall good agreement. In addition, long-term (i.e., 36 h) functionality was assessed in 3 replicates by programming IVG devices (n = 2) to deliver four 2.0-mL doses every 12 h from 2 reservoirs. Devices were programmed to release the first 2 doses from reservoir 1 and the last 2 doses from reservoir 2. The timing and duration of delivery (controlled by the on/off switch) was adjusted for each target dose based on the observed pump release rate (e.g., ∼225 s to deliver 2.0 mL). The cumulative amount released was continuously recorded (i.e., per second) during the 36-h period. The timing of each episode of fluid release was captured by a log from the scale software and observed in person at the scheduled time of release. For the long-term assessment, we programmed IVG devices (n = 2) to release 1 dose (2 mL) of distilled water at time 0, 12, 24, and 36 h. Result are shown in Figure 2C. In support of our hypothesis, both devices were able to accurately (<5% average error) release 2.0 mL of distilled water from each reservoir as programmed. Although both reservoirs were loaded with the same hormone for the in vivo test, our goal was to demonstrate the ability of the device to release fluid from more than one reservoir.

**Figure 2:**
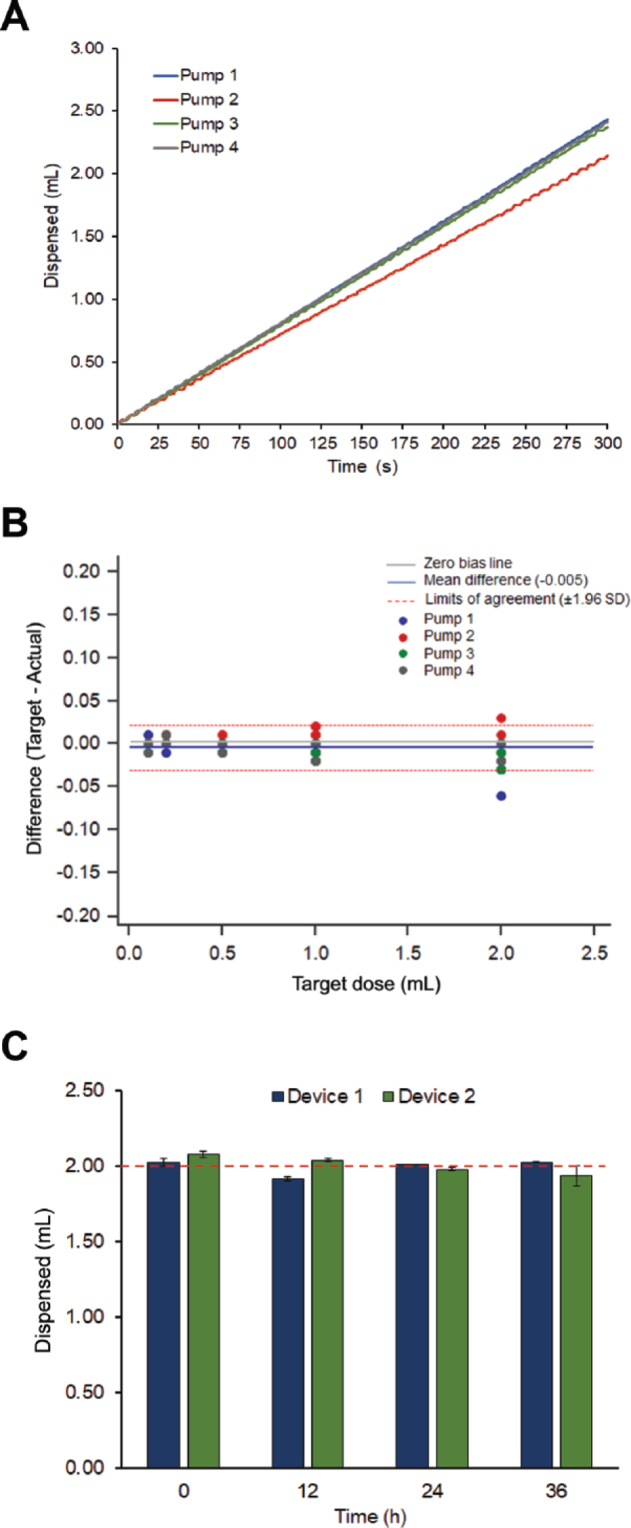
(A) Representative delivery curves of 4 pumps showing amount (mL) of distilled water delivered over a 300-s period. (B) Bland-Altman plot, with differences between target and delivered dose plotted against target dose. Pumps (n = 4) were programmed to release different target volumes (0.1, 0.2, 0.5, 1.0, and 2.0 mL) in 4 replicates (n = 80 doses). The mean difference (−0.005 mL) is represented by the solid blue line and the 95% confidence limits by the red dashed lines. The solid gray line represents the zero-bias line (i.e., 0 mL). (C) Graph depicting average amount (mL) delivered by 2 intravaginal devices programmed to release four 2.0-mL doses (target dose; red dashed line) following a scheme of 1 dose every 12 h. Error bars represent the standard error of the mean

To assess IVG device performance in vivo, lactating nonpregnant primiparous and multiparous Holstein cows from the dairy unit of the Cornell University Ruminant Center (Harford, NY) were enrolled in an experiment conducted from October 2019 to November 2019. Cows were housed in freestall barns with concrete flooring, self-locking head gates, and fans and sprinklers in the feedline. Cows were milked thrice daily at approximately 8-h intervals and were fed a TMR once a day with ad libitum access to feed and water. All procedures performed with cows were approved by the Animal Care and Use Committee of Cornell University (Ithaca, NY).

All cows enrolled received a GnRH treatment (200 μg of gonadorelin acetate given i.m., Gonabreed, Parnell Pharmaceuticals, Overland Park, KS) at 40 ± 3 DIM. Seven days later, transrectal ultrasonography of the ovaries was performed using a 7.5-MHz linear array transducer (Ibex Pro; E. I. Medical Imagining, Loveland, CO). Cows with ≥1 corpus luteum (**CL**) ≥15 mm in diameter (n = 16) were randomly allocated to 1 of 3 treatments: **IM-PGF** (n = 6), **DEV-PGF** (n = 6), and **DEV-CTRL** (n = 4). Cows in IM-PGF received 2 treatments of 25 mg of PGF_2α_ (12.5 mg/mL of dinoprost tromethamine; Lutalyse HighCon, Zoetis, Kalamazoo, MI) 24 h apart as a 2-mL injection in the semimembranosus or semitendinosus muscle. Cows in DEV-PGF received 1 IVG device preloaded with 5 mL of PGF_2α_ in each reservoir and programmed to automatically release 4 doses (i.e., 2 per reservoir) of 25 mg of dinoprost (2 mL per dose) of PGF_2α_. The device was programmed to release the first dose within 2 h of being powered on, coincident with the first blood sample collection (sampling schedule described below), the second dose at 10 h after the first dose, and the third and fourth doses were programmed to be released exactly 12 h after the previous dose. The 2-h interval until release of the first dose was set to allow sufficient time to place all devices (i.e., ∼45 min), whereas the 10-h interval to the second dose allowed us to match the time of each PGF_2α_ release with blood sample collection. Cows in the DEV-CTRL treatment received an IVG device without PGF_2α_ to serve as a placebo control for the presence of the device in the vagina. All devices were removed at 48 h after insertion.

Before device insertion, the vulva and perineal area were washed and disinfected with chlorhexidine solution and dried off with paper towels. Thereafter, vulvar labia were manually opened by one technician while another technician inserted the device using a custom-built applicator. Before device insertion and after removal, a vaginal integrity score (0 = no visible lesions, 1 = superficial lesions, and 2 = erosions of the vaginal mucosa; Walsh et al., 2008) and a mucus score (0 = clear or no mucus, 1 = mucus with flecks of pus, 2 = exudates containing <50% of pus, and 3 = exudates containing ≥50% of pus; Sheldon, 2004) were determined for each cow using a vaginal speculum. In addition, signs of vaginal discomfort (i.e., vaginal straining and back arching) and major changes in behavior [i.e., depressed attitude (i.e., head and ears down, facial distortion), restlessness, recumbency, and vocalization] were assessed by the researchers at the time of each experimental intervention. No scoring system was used for signs of vaginal discomfort or abnormalities in behavior.

Blood samples (∼8 to 9 mL) were collected at time 0 and at 12, 24, 36, 48, and 72 h after treatment by puncture of caudal blood vessels using heparinized evacuated tubes (BD Vacutainer; Becton Dickinson and Co., Franklin Lakes, NJ). Samples were centrifuged at 2,000 × *g* for 20 min at 4°C. Plasma aliquots were harvested and stored at −20°C until assayed for progesterone (**P4**) in duplicate in 3 radioimmunoassays performed as described in Beam and Butler (1997). The average intra-assay coefficient of variation was 13.2% and the interassay coefficient of variation was 17.0%. For this experiment, the presence of a functional CL was defined as circulating P4 ≥1 ng/mL (Ginther et al., 2010), whereas complete CL regression was defined as P4 <1 ng/mL 72 h after treatment.

The effect of experimental treatments on P4 concentrations was evaluated by ANOVA with repeated measures using the MIXED procedure of SAS (version 9.4; SAS institute Inc., Cary, NC) with a model that included treatment, time, and their interaction as fixed effects; cow within treatment was included as a random effect. In addition, cow within treatment was the subject of repeated-measures analysis using a spatial power covariance structure to adjust for the varying time intervals at which blood was collected. Results are presented as LSM ± SEM. Significance was declared at *P* < 0.05.

At the time of device insertion, all cows presented either clear or no vaginal mucus (mucus score = 0), with no visible lesions of the vaginal mucosa (vaginal integrity score = 0). At the time of device removal, 4/10 cows presented mucus with flecks of pus (mucus score = 1) and 5/10 cows presented mild irritation of the vaginal mucosa (vaginal integrity score = 1). Irritation seemed to be located in areas where plastic elbows protruding from the device were in contact with the vaginal mucosa. Nevertheless, none of the cows had a score of 2 or erosions of the vaginal mucosa. In addition, none of the cows presented noticeable signs of vaginal discomfort or major abnormalities in behavior as defined for this experiment. All IVG devices (10/10) remained in situ for the 48-h study period (100% retention rate). Hormone reservoirs for all devices recovered from cows in the DEV-PGF group contained approximately 1 mL of fluid at the time of removal, suggesting release of the approximate amount of fluid programmed to be released per reservoir (i.e., 4 mL).

The effect of treatment on circulating P4 concentration profiles is presented in Figure 3. There was an effect of treatment (*P* = 0.003), time (*P* < 0.001), and an interaction between treatment and time (*P* = 0.003). From 24 to 72 h, cows in the DEV-PGF and IM-PGF treatments had lesser concentrations of P4 than cows in the DEV-CTRL treatment (negative control). In contrast, concentrations of P4 did not differ for the DEV-PGF and IM-PGF treatments during the entire sampling period. Cows treated with PGF_2α_ had a 67 to 92% reduction in concentrations of P4 by 36 h after the first treatment. Except for one cow from the DEV-PGF (P4 = 1.20 ng/mL) and one cow from the IM-PGF treatment (P4 = 1.21 ng/mL), all cows had P4 <1 ng/mL at 36 h after treatment. Thereafter, concentrations of P4 continued to decline until the end of the sampling period (0.14 to 0.50 ng/mL) when, except for one cow from the IM-PGF treatment (P4 = 0.50 ng/mL), all cows had P4 concentrations <0.5 ng/mL. In contrast, cows in the DEV-CTRL treatment did not experience a decline in P4 concentrations at any point after device insertion and average P4 concentration for the group was never below 1 ng/mL.

**Figure 3:**
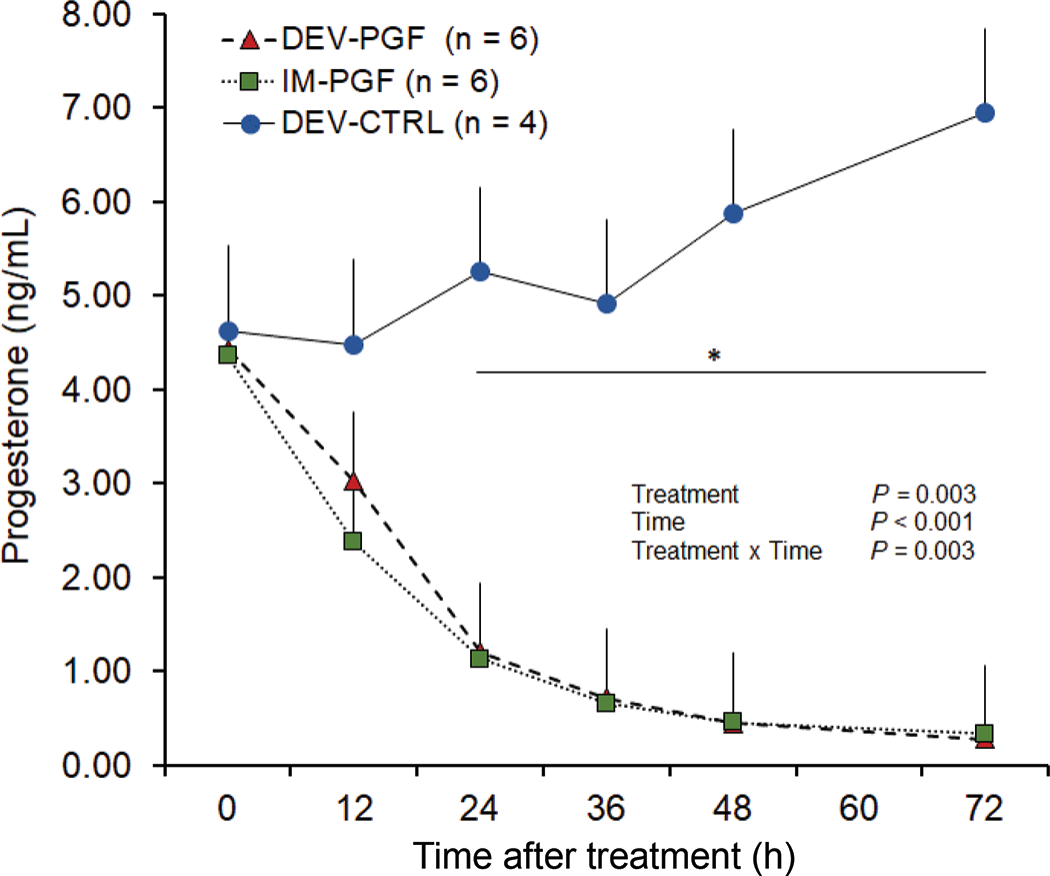
Circulating concentrations of progesterone (P4) from 0 to 72 h after application of treatments. Lactating Holstein cows with at least 1 corpus luteum ≥15 mm in diameter were randomly allocated to 1 of 3 treatments: (1) IM-PGF (n = 6): two 25-mg i.m. doses of PGF_2α_ 24 h apart; (2) DEV-PGF (n = 6): four 25-mg doses of PGF_2α_ released automatically by the intravaginal device every 12 h; and (3) DEV-CTRL (n = 4): insertion of an intravaginal device with no PGF_2α_ (placebo control). Values are presented as LSM ± SEM. *Circulating concentrations of P4 differed from 24 to 72 h after treatment as cows in IM-PGF and DEV-PGF had lesser P4 than cows in the DEV-CTRL treatment.

In the present study, we developed and evaluated a programmable, reusable IVG device for controlled hormone delivery in cattle. The current prototype device is capable of automatically delivering up to 2 different hormones at predefined time points. Despite a minor increase in variability between target and delivered dose observed with increasing amounts of target doses, in vitro results showed that the current device permits precise control of released hormone amount and timing, and that performance does not seem to decrease over time (at least for up to 36 h). This is relevant because causing the desired biological response with reproductive hormones such as PGF_2α_ and GnRH depends on hormone release in the right amount at the right time with periods of several days in between treatments. Although our proof-of-concept results with PGF_2α_ were encouraging, additional research is required to test the ability of the developed device to release more than one hormone, following typical schedules of synchronization of ovulation protocols for TAI.

In support of our hypothesis, the device was able to automatically deliver PGF_2α_ and induce complete luteal regression in lactating dairy cows. Our reasoning to include 4 doses of 25 mg of PGF_2α_ for cows in the DEV-PGF group was to ensure that luteal regression occurred if the device was capable of releasing PGF_2α_. This was a first proof-of-concept experiment designed to evaluate many aspects of device functionality, retention, and hormone release. The differences in P4 profiles with the placebo controls and the lack of significant differences with IM-treated cows suggested that CL regression was caused by the PGF_2α_ released by the device rather than the potential physical effect of the presence of the device in the vagina. These results were expected because we recently demonstrated similar P4 profiles and luteal regression risk in cows that received PGF_2α_ through the IVG or IM route of administration (Wijma et al., 2016; Masello et al., 2020) and there are no obvious biological reasons to expect that the presence of the device in the vagina would cause CL regression. Additional research is needed to determine the optimal dose, frequency, and timing of PGF_2α_ to achieve CL regression rates similar to those observed with IM injection of PGF_2α_ because the purpose of this first experiment was to provide proof-of-concept of automated PGF_2α_ release.

There are many concerns with electronics, packaging, retention, and cow comfort. One concern is the potential detrimental effect of moisture and temperature on components of an electronic device for vaginal insertion (Cross et al., 2004). In the current experiment, the PCB was conformal coated, the device casing was sealed, and extra protection was provided by the external silicone rubber coating. Thus, although this could not be confirmed by observing timing and amount released for each programmed dose, we speculate that the device functioned properly as evidenced by the release of most of the PGF_2α_ loaded in the device reservoirs. If major malfunction occurred, there were 2 main scenarios were possible. In one scenario, the board would have shut down and no hormone would have been released. The other scenario would have been the opposite, with pumps running continuously until all battery charge was consumed. In the latter case, no PGF_2α_ would have been left in the reservoirs, which was not the case, and we confirmed that charge remained in the battery. One of the limitations of the current experiment, however, was that the exact timing of PGF_2α_ delivery and the amount released at each time point could not be confirmed because the current version of the device was not designed to communicate with external sources. In vivo monitoring of the timing of hormone release would have required device removal and reinsertion multiple times. This was avoided because of the potential irritation of the vaginal mucosa and cow discomfort due to repeated insertion and removal. Thus, future versions of the device will include wireless communication to enable real-time in situ monitoring of hormone release.

Proper device design for maximal retention and cow comfort is also relevant. During the in vivo test, all devices were retained for at least 2 d. In addition, we did not see attempts from cows to expel the device from the vaginal cavity. We observed vaginal discharge with flecks of pus in approximately half the cows that received a device. These findings were expected because it is known that insertion of IVG P4-releasing devices such as the PRID and CIDR-B might result in mucopurulent vaginal discharge in a substantial proportion of cows (Chenault et al., 2003; Walsh et al., 2007; von Krueger and Heuwieser, 2011). Nevertheless, in previous studies, the observed vaginal discharge seemed to be inconsequential for fertility (Walsh et al., 2007; von Krueger and Heuwieser, 2011). In the current experiment, we also observed mild irritation of the vaginal mucosa in half the cows with an IVG device. We speculate this was caused by the plastic elbows protruding from the device because irritation was highly localized in small areas of the vaginal mucosa that may have been in contact with these elements of the device. Thus, future device optimization will focus on minimizing vaginal irritation.

Results from this proof-of-concept experiment, which demonstrated the feasibility of automated delivery of PGF_2α_ and successful induction of complete CL regression in lactating dairy cows, are encouraging. Nevertheless, before automated electronically controlled IVG hormone-releasing devices can be deployed for control of reproduction in cattle, additional research is necessary to characterize device performance and implementation in detail. In particular, future refinements include demonstrating the ability of the device to release more than one hormone of interest (e.g., GnRH, PGF_2α_, and P4) for a longer period (e.g., ∼10 to 20 d) and following more complex drug delivery profiles. Once optimized, these intravaginal hormone-delivery devices may be an alternative to the injection methods presently used to administer hormones for synchronization of ovulation. On-farm use of this automated delivery system might simplify herd management, reduce animal disruption, and enable the development of improved synchronization of ovulation protocols.
