# Erratum to “An automated controlled-release device for intravaginal hormone delivery” (JDS Commun. 1:15–20)

**DOI:** 10.3168/jdsc.2022-3-2-161

**Published:** 2022-03-16

**Authors:** M. Masello, Y. Ren, D. Erickson, J.O. Giordano

In the Notes section (page 20), the conflicts of interest statement was missing. The following statement should be added: **The authors have not stated any conflicts of interest.**

## References

[bib1] Masello M., Ren Y., Erickson D., Giordano J.O. (2020). An automated controlled-release device for intravaginal hormone delivery. JDS Commun..

